# Child–Adult Contract for Prevention of Tobacco Use: “As-Treated” Analysis of a Cluster Randomized Controlled Trial (the TOPAS Study) at 3-Year Follow-Up

**DOI:** 10.1007/s11121-023-01598-y

**Published:** 2023-10-25

**Authors:** Dorien Tecla Beeres, Anni-Maria Pulkki-Brännström, Maria Nilsson, Maria Rosaria Galanti

**Affiliations:** 1 Department of Global Public Health, Karolinska Institutet, Tomtebodavägen 18A, 17177 Stockholm, Sweden; 2 Department of Public Health, Erasmus MC, Rotterdam, The Netherlands; 3 Department of Epidemiology and Global Health, Umeå University, Umeå, Sweden; 4 Centre for Epidemiology and Community Medicine, Stockholm, Sweden

**Keywords:** Adolescence, Tobacco use, As-treated analysis, School-based prevention

## Abstract

To estimate the effect of a 3-year commitment to remain tobacco free on tobacco uptake among high school students in Sweden. The commitment is developed in the form of a contract between a child and a significant adult, constituting the core component of Tobacco-free Duo (T-Duo), a Swedish school-based tobacco prevention program. Secondary analysis of data from a cluster randomized controlled trial. Participants were 586 students in high schools assigned to the intervention arm of T-Duo. At inception, participants attended grade 7 (i.e., age 12–13). Only students who were tobacco naïve at baseline for the respective outcome and participated in all follow-ups were included. The exposure was defined as signing a 3-year contract with a significant adult, categorized as “stable contract” (3 years contract with the same contract partner), “unstable” (signed a contract sometime during follow-up but this was not sustained over time and/or with the same partner), and “no contract” at all during the intervention period. The primary outcome was having never tried cigarette smoking at the end of grade 9. Exposure and outcomes were self-reported in yearly questionnaires. Of 586 students, 321 (55%) held a stable contract, 204 (35%) an unstable contract, and 61 (10%) did not sign a contract at all. At the end of grade 9 (age 15–16), the relative risk (RR) to remain cigarette free was 1.11 (95% CI 1.00–1.22) (Number Needed to Treat = 10) among students in any type of contract compared to students that did not write a contract at all. The RRs for remaining tobacco free (secondary outcomes) ranged from 1.07 (0.98–1.16) for regular snus use to 1.16 (1.00–1.35) for any type of tobacco use. A commitment to remain tobacco free through a child–adult contract seems to exert a preventive effect on the uptake of tobacco use among Swedish adolescents over 3 school years. The current findings apply to a selected sample of both schools and students. Registration: Current Controlled Trials ISRCTN52858080 Date: January 4, 2019, retrospectively registered.

**Supplementary Information:**

The online version contains supplementary material available at 10.1007/s11121-023-01598-y.

## Introduction

Several smoking prevention interventions targeting youths propose as a distinctive characteristic a public commitment to remain smoke free, either between individuals or within groups such as school classes. The assumption that a commitment may prevent or delay the onset of smoking is based on theories of social influence and correction of norms (Isensee & Hanewinkel, 2012). Social influences through one’s micro-environment are identified as strong determinants for smoking initiation in adolescence, for example, through smoking friends or parents (Hefler et al., 2017). The impact of parents on smoking norms and smoking uptake is strong, at least in early adolescence (Hefler et al., 2017). The commitment to remain tobacco free in form of a formal contract between an adolescent and a significant adult may reinforce the positive social influence and may entail positive reward when committing to the agreed behavior (Flay, 2009; Isensee & Hanewinkel, 2012; Thomas et al., 2015).

A widely implemented and evaluated intervention that is based on a shared commitment to remain tobacco free is the Smoke-Free Class competition (SFC). SFC entails a commitment to be a non-smoking class for a set period, often 6 months. Pupils sign both a class contract and an individual contract as commitment not to smoke during the competition (Institute for Therapy and Health Research, 2009). SFC started in 1989 in Finland, and since then disseminated throughout several middle- and high-income countries (Isensee & Hanewinkel, 2012). The European Union commissioned the dissemination of SFC and provided financial support for its implementation (Institute for Therapy and Health Research, 2009). From 1997 to 2008, interventions similar to SFC were widely implemented in more than 20 countries in Europe and overseas, e.g., the German program “Be Smart—Don’t Start” (Isensee & Hanewinkel, 2018), the Canadian “Mission TNT.06” (Kairouz et al., 2009), the Danish “X:IT study” (Andersen et al., 2015), and the Czech “Our Class Does Not Smoke” (Hrubá et al., 2007). Previous evaluations of these programs in randomized trials showed mixed results (Andersen et al., 2019; Hrubá et al., 2007; Isensee et al., 2012), while a meta-analysis that summarized the results from several European versions of the SFC found a 14% lower risk of current smoking among students participating in SFC compared to students that did not participate in the intervention (Isensee & Hanewinkel, 2012).

The large majority of these SFC evaluations were based on the “intention-to-treat (ITT)” analysis in randomized trials, which is the recommended primary analysis. ITTs are known to minimize bias due to confounding and intend to measure the effect of the intervention when delivered as intended on a population level (Hernán & Hernández-Díaz, 2012). However, the results of ITTs tend to underestimate a true effect due to imperfect implementation, i.e., they rather measure the effect of being assigned to the intervention (Hernán & Hernández-Díaz, 2012). In the context of multi-component interventions, it has been proposed to assess the effect of individual components in an “as-treated analysis” complementary to a formal ITT analysis (Hernán & Hernández-Díaz, 2012; Institute for Therapy and Health Research, 2009).

Despite recommendations to evaluate individual components, there are very few studies that assessed the effect of the core component of SFC, i.e., the agreement between students within a class to remain tobacco free for a set time period (Bast et al., 2021). A Danish study assessed predictors for participation in the contract (Bast et al., 2021).

The Swedish intervention “Tobacco-free Duo (T-Duo)” is a self-standing intervention developed in Sweden in the early 1990s. T-Duo consists of a school-based program including six components, where the core component is represented by a mutual agreement to remain tobacco free, stipulated between a student and his/her caregiver or another adult with whom he/she holds a close relation based on trust and support, e.g., other family member or school nurse (Galanti et al., 2020). In contrast to the European SFC where the contract covers a 6-month period and was carried out and evaluated as a class activity, the T-Duo contract foresees a 3-year commitment between two individuals (student-adult pair) and includes all types of tobacco (Galanti et al., 2020). In an earlier evaluation of the T-Duo program according to an intention-to-treat (ITT) analysis, we found a relative risk to remain cigarette free over a 2-year period of 1.03 (0.98–1.08) among students assigned to the T-Duo compared to students in the control group (Beeres et al., 2022).

We conducted a secondary “as-treated” analysis to estimate the effect of the adherence to the core component of the intervention (i.e., the contract between an adolescent and significant adult to remain tobacco free) during the entire 3-year study period. In contrast to a more “classical” as-treated analysis, the purpose of evaluating a specific component is slightly different; in that, we aim to estimate the effect of a particular component, namely the public commitment to remain smoke free, among students who were assigned to the whole intervention.

We hypothesized that the probability to have refrained from using cigarettes (and/or other tobacco) at the end of the 9th grade would be highest among adolescents that built a stable tobacco-free pair with the same adult partner for 3 years, lowest among adolescents who did not enter a contract at all, with adolescents partially adhering during 3 years in an intermediate position.

The research questions were formulated as follows: (1) Is the probability of having refrained from using cigarettes/any tobacco by the end of the third follow-up higher among adolescents who were in a tobacco-free pair compared to adolescents who were not in a pair and (2) was there a gradient in the probability of remaining tobacco free between adolescents who were in a continuous and stable partnership, those who managed to hold a contract for just a part of the period or changed partner and those who did not sign the contract at all?

## Method

### Study Design and Data

This is a secondary analysis using data from a cluster randomized controlled trial (c-RCT, the TOPAS study; cluster level = schools, unit of analysis = individual students). Randomization occurred at school level (ratio 1:1) (Galanti et al., 2020).

Schools were eligible for participation if they had at least two parallel classes in grade 7 and if they did not earlier implement the Tobacco-free Duo program. A total of 571 eligible schools were randomly selected and invited to participate in the project, of which 43 (8%) were enrolled (Galanti et al., 2020). Schools that declined participation were more often publicly run, located outside the Stockholm Region, had a higher average number of students and a lower proportion of teachers with university education compared to participating schools (Beeres et al., 2021). All students enlisted in grade 7 (about 13 years of age) of the participating schools were eligible and invited to participate in the study. Explicit informed consent to data collection, analysis, and reporting was asked from the participating schools and students’ caregivers prior to inclusion in the study (Galanti et al., 2020).

Smoking, tobacco use, and contract status were self-reported in a paper questionnaire, administered at baseline and at the end of each school year among participating adolescents. Participants were recruited in 7th grade (fall 2018) when they took part in the baseline assessment. Three follow-up waves were conducted, the first of which took place in May/June 2019 (end of grade 7), the second in May/June 2020 (end of grade 8), and the third one in May 2021 (end of grade 9). Questionnaires were completed at school or, if the adolescent had changed schools, sent to the home address and took approximately 10–20 min to complete. Information on caregivers’ characteristics and substance use was collected through caregivers’ questionnaires administered at the same time points. Information on school characteristics was collected from a public database (Skolverkets Internetbaserade Resultat- och kvalitetsInformationsSystem) (SIRIS) from the Swedish National Agency for Education. Information about implementation of the intervention and the conduct of other prevention or health promotion programs was self-reported by each school’s staff through annual web questionnaires.

### Participants

The study population consisted of students in the T-Duo intervention arm of the c-RCT. Only students with valid questionnaire reports for all three assessments were included (*n* = 586).

### Intervention

T-Duo is a multi-component school-based intervention with the aim to prevent adolescents from tobacco initiation. The intervention consists of six components with the tobacco-free pair as core component. The tobacco-free pair refers to an agreement between an adolescent and a significant adult (at least 18 years old) of the young person’s choice, in the beginning of grade 7. Together they commit to remain tobacco free at least during the following 3 years (until the adolescent leaves compulsory school at about 15 years of age), during which time the adult is expected to actively support the adolescent to keep the commitment. The agreement takes the form of a formal contract signed by the adolescent and the significant adult.

The remaining five components include student information (school informs students about tobacco and the tobacco-free pair and invites them to sign the contract), parent information (school informs parents about tobacco and how they can support adolescents to remain tobacco free), membership card for adolescents who sign a contract (entitles to fringe benefits arranged by the school), yearly disclosure of tobacco-free status (signed by both contract partners) that entitles adolescent to participate in a prize draw at school, and four structured classroom education lessons per school year (age adapted and interactive). A detailed description of the intervention can be found in the published study protocol (Galanti et al., 2020).

The exposure in this study was defined as building a contract, with a primary categorization into ever signed a contract vs never (reference). In a secondary categorization, ever signing a contract was further divided into stable contract (1) and unstable contract (2), as defined below. In theory, all students in the schools randomly assigned to the T-Duo program were targeted to sign the contract. In practice, whether a student signed and sustained a contract was dependent on implementation at the school level and on the relationship between each student and the chosen significant adult.

### Exposure

#### Contract Status

Ever contract: All students who signed a contract at least once during follow-up.

Stable contract (1): Students were considered as being in a stable contract if they reported to have a valid signed contract at each follow-up assessment with the same adult partner during a 3-year period

Unstable contract (2): Unstable contract refers to students that reported (i) having signed the contract during some but not all 3 years and/or (ii) having changed contract partner.

No contract (reference): Students that reported not having signed a contract at any follow-up assessment. The item and response options can be found in Appendix 2.

### Outcome

#### Primary Outcome

Having never tried smoking (not even a few puffs) at follow-up 3 (yes/no) among never users at baseline, using the question: “(1) “Have you ever tried smoking a cigarette, even if it was just a few puffs?” “No, never tried; Yes.”.

#### Secondary Outcomes

Never smoked a whole cigarette; never smoked regularly; never tried other types of tobacco use, i.e., smokeless tobacco (snus) use, e-cigarettes and water pipe, and a composite outcome defined as never used any of these four tobacco products at follow-up 3. All outcomes were self-reported based on questionnaires, the items and response options of which can be found in Appendix 2.

### Covariates

#### Student-Level Covariates

In line with a prespecified directed acyclic graphs (DAG) (Appendix 3), we included as covariates for control of confounding students’ sex and smoking friends (having at least one friend that smokes regularly), parental education, parental country of birth, and current parental smoking, and snus use.

Direct measures of student-level connection to school were not available. It is known that within peer associations, values and attitudes are shared to a substantial extent (Ilmarinen et al., 2017). As proxy for student-level connection to school, responses to the question on “how many of the friends with whom you spend most of your time enjoy school/perform well at school” were used. For the corresponding questionnaire items see Appendix 2.

#### School-Level Covariates

We included size of the school, teacher density (ratio teacher: student), proportion of teachers with university education, students’ average merit score, and the presence of other prevention programs and health promotion programs.

#### School-Level Implementation

School-level implementation of the full T-Duo intervention was categorized as “Per Protocol”, “Satisfactory,” or “Poor” based on whether the school implemented each component at the intended time. The classification was previously used in a published report and based on the theoretical assumption that complete implementation would have the largest effect but also a practical concern for sufficiently even-sized groups. Per protocol means that student and parent information were given in year 7; tobacco-free contracts were signed in year 7; there was disclosure of tobacco-free status and a prize draw at the end of each academic years 7, 8, and 9; and structured classroom education at least twice for each class during years 7, 8, and 9. Satisfactory means that three or four of these components were completed at the intended times. Poor means that none; one or two components were carried out according to the protocol. Implementation of membership cards was not included in this classification because most schools reported big difficulties to attract fringe benefits early on and therefore did not issue membership cards (Nilsson et al., 2022).

### Statistical Methods

Multilevel generalized linear regression models with the Poisson family and log link were used to calculate the probabilities (risk ratios and 95% confidence intervals) to have remained a non-smoker/non-tobacco user at the third follow-up among students who entered a contract at all, subsequently subdivided into (1) a stable contract or (2) an unstable contract, compared to students who did not sign a contract at all (reference category). Only students who had never tried the corresponding type of tobacco prior to baseline and with complete information were included in the analyses. First, we computed the unadjusted model (model 0), followed by a model with adjustment for individual covariates only (model 1), and a final model adjusted for both school-level and individual-level covariates (model 2). To assess the proportion of variation in tobacco outcomes accounted for by clustering in schools, we computed the intra-class correlation (ICC) of the empty model for each tobacco outcome.

For all outcomes of the final model (model 2), we computed the Bayes factor (BF_10_). As prior (*θ*), the pooled risk ratio of 0.86 (95% CI 0.79–0.94) as reported in the meta-analysis by Isensee and Hanewinkel (2012) was used, transformed to the risk to remain a non-smoker using the raw data (RR = 1.10) (Isensee & Hanewinkel, 2012). A half-normal distribution with the log of the transformed pooled effect size as standard deviation was used to represent the alternative hypothesis (Dienes, 2014). In addition, to assess clinical significance, we computed the Number Needed to Treat (NTT) to prevent one adolescent from smoking (NTT = 1/absolute risk reduction).

Three sensitivity analyses were performed. (1) To assess the effect of exclusion of students with incomplete information, we repeated the main analysis including students that answered at baseline and follow-up 3 (contract status based on status end of year 3). Since only 20 students had missing covariates in model 2 compared to model 0, we did not perform any sensitivity analysis to account for missing information. (2) To compare the estimates of this as-treated analysis with the effect estimates of the published ITT analysis at 2-year follow-up, we did an interim analysis at 2-year follow-up. (3) Finally, to test whether there was a gradient in the effect of contract writing, we used multilevel mixed-effects ordered logistic models (ordinal family and logit link) to calculate the odds ratio of remaining tobacco free. R version 3.6.0 and STATA version 15.1 were used for the statistical analysis. Command syntax for all analyses can be found at the open science framework (https://osf.io/e8nbd/).

## Results

### Baseline Characteristics

Of the 34 participating schools in the TOPAS study, 17 schools took part in the T-Duo intervention, of which 16 were still actively participating after 3 school years. All 1477 students of the 17 intervention schools were invited to participate of which 906 were granted guardians’ consent. A total of 840 students of the T-Duo schools taking part answered the baseline survey, of which 586 (70%) also answered at all three follow-up points and were included in this study.

Of the 586 students constituting the analytical sample, 321 (55%) held a stable contract; 204 (35%) students entered an unstable contract, because they either switched contract partner (*n* = 18) or wrote the contract for only 1 or 2 years (*n* = 186); and 61 (10%) did not sign a contract at all. Students who were in a stable contract were more likely to be female, to have at least one foreign-born parent, to have parents without university degree, and less likely to have smoking friends compared to students who were in an unstable contract or did not write a contract at all (Table 1). The majority of students who signed a contract did so with their parent (88%), while 12% signed a contract with someone else, for example, a sibling, “bonus parent” or the school nurse and this did not differ substantially between the stable and unstable contract groups. Students who signed a contract for all 3 years but had missing information on contract partner were classified as being in a stable contract (*n* = 1). Students who were not retained were more likely to have already tried tobacco at baseline (*n* = 13, 5.0%) compared to students who participated in all four surveys (*n* = 9, 1.5%) (Table 1). Smoking friends and school-level implementation of the six T-Duo components were also related to retention status (Table 1).

**Table 1:** Baseline characteristics of students enrolled in T-Duo intervention schools, by contract and retention status

	**Analytical sample (complete cases) (*N* = 586)**			**Retention status (*N* = 840)**	
	**No contract (*n* = 61)**	**Unstable contract (*n* = 204)**	**Stable contract (*n* = 321)**	**Not complete**^**3**^** (*n* = 254)**	**Complete **^**3**^** (*n* = 586)**
	*n* (%)^5^	*n* (%)^5^	*n* (%)^5^	*n* (%)^5^	*n* (%)^5^
**Gender**					
Female	22 (37)	96 (47)	177 (55)	124 (49)	295 (51)
Male	36 (60)	106 (52)	139 (43)	121 (48)	281 (48)
Other	2 (3)	2 (1)	4 (1)	7 (3)	8 (1)
**Caregiver’s age at baseline** *mean (SD)*	45.2 (5.5)	45.1 (6.0)	44.4 (5.0)	44.3 (6)	44.7 (5)
**Caregivers’ highest achieved education**					
Both without university degree	9 (17)	52 (30)	64 (21)	52 (25)	125 (24)
At least one with university degree or equivalent	45 (83)	120 (70)	236 (79)	152 (75)	401 (76)
**Caregivers’ current employment status**					
Both caregivers employed	49 (92)	165 (93)	283 (94)	181 (89)	497 (93)
At least one caregiver unemployed	4 (8)	12 (7)	19 (6)	22 (11)	35 (7)
**Caregivers’ country of birth**					
Both born in Sweden	49 (92)	128 (75)	239 (82)	152 (76)	416 (81)
At least one born outside of Sweden	4 (8)	42 (25)	54 (18)	49 (24)	100 (19)
**Caregivers’ tobacco use**					
Any current cigarette use (yes)	5 (9)	15 (8)	15 (5)	21 (10)	35 (7)
Any current snus use (yes)	6 (13)	13 (9)	23 (9)	16 (9)	42 (9)
**Student’s tobacco use (ever)**					
Lifetime cigarettes (yes)	3 (5)	5 (2)	1 (0)	13 (5)	9 (1.5)
Lifetime snus (yes)	2 (3)	8 (4)	6 (2)	14 (6)	16 (3)
Lifetime e-cigarettes (yes)	6 (10)	16 (8)	11 (3)	39 (16)	33 (6)
Lifetime water pipe (yes)	0 (0)	5 (2)	3 (1)	12 (5)	8 (1)
Lifetime any smoking^4^ (yes)	7 (11)	22 (11)	18 (6)	48 (18)	47 (8)
**Close friends who smoke regularly (> 1/week)**					
None	55 (92)	157 (82)	297 (95)	194 (81)	509 (90)
At least one	5 (8)	35 (18)	15 (5)	45 (19)	55 (10)
**Friends that enjoy school**					
Half or less	34 (58)	127 (63)	295 (64)	51 (61)	366 (63)
More than half	25 (42)	76 (37)	114 (36)	32 (39)	215 (37)
**Friends that perform well at school**					
Half or less	19 (33)	66 (33)	103 (32)	33 (41)	188 (33)
More than half	39 (67)	67 (67)	214 (68)	48 (59)	386 (67)
**Contract partner**^**2**^					
Parent	NA	74 (82)	288 (90)	92 (88)	365 (88)
Other	NA	16 (18)	32 (10)	13 (12)	48 (12)
**School-level variables**					
Average merit score *mean* (*SD*)	232 (24)	228 (18)	227 (17)	237 (23)	228 (18)
% teachers with university education *mean* (*SD*)	75% (12)	73% (17)	72% (17)	70% (19)	73% (16)
Students per teacher *mean* (*SD*)	12.0 (1.6)	12.6 (1.8)	12.0 (1.7)	12 (1.6)	12 (1.8)
% foreign students *mean* (*SD*)	18% (10.5)	22% (9.7)	20% (9.7)	20% (9)	20% (10)
Health promotion program^1^ (yes)	6 (10)	53 (26)	55 (17)	70 (28)	114 (19)
Other prevention program^1^ (yes)	4 (7)	39 (19)	22 (7)	28 (11)	65 (11)
**Implementation entire T-Duo**					
Poor	20 (33)	50 (25)	68 (21)	80 (32)	138 (24)
Satisfactory	34 (56)	111 (54)	156 (49)	134 (53)	301 (51)
Per protocol	7 (12)	43 (22)	97 (30)	40 (16)	147 (25)

^1^Derived from school-based web questionnaires

^2^Information on contract partner was missing for 91 (60%) of the students in an unstable contract

^3^Incomplete retention means non-participation at follow-up in one or more occasions. Complete retention implies participation in all follow-up occasions

^4^Any type of smoking includes cigarettes, e-cigarettes, and/or water pipe

^5^Mean (SD) instead of *n* (%) for continuous variables

The relative risk (RR) to remain tobacco free was higher among students in a contract compared to the students without a contract at all for all outcomes (RR ranges [1.07–1.16]) (Table 2). The adjusted RR to have remained a non-cigarette smoker (not even a few puffs, primary outcome) over the 3-year period was 1.11 (1.00–1.22) among students that formed a tobacco-free pair for at least 1 year compared to students without a contract at all. In terms of absolute risk difference (ARD), the percentage of students that remained cigarette free at the end of follow-up was 10.0% (95% CI 0.00–20.0) higher among students in a contract compared to students without a contract at all, corresponding to number needed to treat (NNT) of 10 (Appendix 1, Table 4). For the secondary outcomes, the adjusted RRs to remain tobacco free at the end of follow-up ranged from 1.07 (0.98–1.16) for regular snus use to 1.16 (1.00–1.35) for any type of tobacco use among students in a contract compared to students without contract at all (Table 2). In absolute terms, this is related to a NNT of 7 for any tobacco use, 13 for e-cigarettes use, and 9 for snus use (Appendix 1, Tables 4 and 5).

**Table 2:** Risk ratios to remain tobacco free at the end of grade 9 among students that were never users of the corresponding type of tobacco at baseline and participated in all three follow-up surveys by contract status (ever vs never signed a contract)

	**Contract status**		**Risk ratio’s (95% CI) to remain a non-user after 2 school years among non-users at baseline**			
Not started with	Never contract (ref) Ever contract^4^ *Proportion non-user’s follow-up 3*	ICC (schools)	Model 0—crude RR (95% CI)	Model 1—adjusted for individual covariates^1^ RR (95% CI)	Model 2—adjusted for individual + school covariates^2^ RR (95% CI)	Bayes factor^3^ BF_10_
**Primary outcome**						
Cig. smoking (even a few puffs**)**	41/54 (76) 427/514 (83)	0.018	1 (ref) 1.09 (1.00–1.20)	1 (ref) 1.12 (1.00–1.26)	1 (ref) 1.11 (1.00–1.22)	- 4.0
**Secondary outcomes**						
Cig. smoking (whole cigarette)	46/53 (87) 469/514 (91)	0.022	1 (ref) 1.05 (0.99–1.12)	1 (ref) 1.09 (1.01–1.18)	1 (ref) 1.10 (1.01–1.21)	- 3.2
Regular cig. smoking	48/54 (89) 505/517 (98)	0.004	1 (ref) 1.10 (0.99–1.22)	1 (ref) 1.10 (0.97–1.25)	1 (ref) 1.10 (0.96–1.27)	- 2.2
Snus use	40/55 (73) 402/506 (79)	0.037	1 (ref) 1.09 (0.93–1.28)	1 (ref) 1.15 (0.94–1.41)	1 (ref) 1.11 (0.94–1.33)	- 1.7
Reg. snus use	50/57 (88) 494/519 (95)	0.000	1 (ref) 1.09 (1.03–1.15)	1 (ref) 1.09 (1.01–1.17)	1 (ref) 1.07 (0.98–1.16)	¨ 2.3
E-cig. use	40/50 (80) 424/492 (86)	0.064	1 (ref) 1.08 (0.99–1.18)	1 (ref) 1.10 (1.00–1.22)	1 (ref) 1.09 (0.97–1.21)	- 1.7
Water pipe	47/53 (89) 481/505 (95)	0.030	1 (ref) 1.07 (0.97–1.19)	1 (ref) 1.12 (0.98–1.27)	1 (ref) 1.12 (0.99–1.28)	- 2.9
Any tobacco use	33/51 (65) 353/483 (73)	0.040	1 (ref) 1.13 (0.99–1.29)	1 (ref) 1.23 (1.03–1.46)	1 (ref) 1.16 (1.00–1.35)	- 2.8

^1^Adjusted for student’s gender, student’s smoking friends, friends enjoying school and performance of friends at school, parental education, parental occupation, and parental smoking

^2^Adjsuted for 1 + school level variables: average merit score, school average parental education, teacher density, proportion of teachers with university education, implementation of other prevention programs, implementation of other health promotion programs, and implementation level of all other T-Duo components using the three categories (poor, satisfactory, per protocol)

^3^Bayes factor less than 1/3 indicates evidence for the 0 hypotheses (no effect), greater than 3 indicates evidence for an effect (alternative hypothesis), and any value between 1/3 and 3 indicates that the findings are inconclusive (Dienes, 2014)

^4^Wrote a contract at least once during the 3-year follow-up

**Table 3:** Risk Ratios to remain tobacco free at the end of grade 9 among students in the T-Duo program who were never users of the corresponding type of tobacco at baseline, by contract status

	**Contract status**		**Risk ratios (95% CI) to remain a non-user after 3 school years among non-users at baseline**			
	**Never contract (ref)** **Unstable contract**^**4**^ **Stable 3-year contract** *Proportion non-users follow-up 3*	**ICC**	**Model 0—crude** RR (95% CI)	**Model 1—adjusted for individual covariates**^**1**^ RR (95% CI)	**Model 2—adjusted for individual + school covariates**^**2**^ RR (95% CI)	**Bayes factor**^**3**^ (BF_10_)
**Primary outcome**						
Cig. smoking (even a few puffs)	41/54 (76) 152/197 (77) 275/317 (87)	0.018	1 (ref) 1.02 (0.91–1.13) 1.14 (1.04–1.25)	1 (ref) 1.06 (0.92–1.22) 1.15 (1.03–1.29)	1 (ref) 1.04 (0.91–1.19) 1.15 (1.04–1.26)	- 0.8 17.2
**Secondary outcomes**						
Cig. smoking (whole cigarette)	46/53 (87) 172/196 (88) 297/318 (93)	0.022	1 (ref) 1.01 (0.93–1.10) 1.08 (1.01–1.15)	1 (ref) 1.06 (0.96–1.17) 1.11 (1.03–1.20)	1 (ref) 1.07 (0.95–1.19) 1.13 (1.03–1.23)	- 1.1 6.7
Regular cig. smoking	48/54 (89) 189/198 (95) 316/319 (99)	0.004	1 (ref) 1.07 (0.96–1.20) 1.11 (1.00–1.23)	1 (ref) 1.07 (0.93–1.23) 1.11 (0.98–1.26)	1 (ref) 1.08 (0.94–1.25) 1.12 (0.98–1.28)	- 1.2 2.1
Snus use	40/55 (73) 147/194 (76) 255/312 (82)	0.037	1 (ref) 1.04 (0.87–1.25) 1.12 (0.95–1.33) 1	1 (ref) 1.12 (0.89–1.42) 1.18 (0.96–1.43)	1 (ref) 1.06 (0.84–1.35) 1.15 (0.97–1.36)	- 1.0 2.4
Reg. snus use	50/57 (88) 182/202 (90) 312/317 (98)	0.000	1 (ref) 1.03 (0.96–1.10) 1.10 (1.03–1.17)	1 (ref) 1.04 (0.96–1.14) 1.11 (1.04–1.19)	1 (ref) 1.02 (0.93–1.12) 1.09 (1.01–1.17)	- 0.4 17.1
E-cig. use	40/50 (80) 149/183 (81) 275/309 (89)	0.064	1 (ref) 1.03 (0.93–1.13) 1.12 (1.00–1.26)	1 (ref) 1.05 (0.94–1.18) 1.15 (1.04–1.27)	1 (ref) 1.01 (0.87–1.19) 1.13 (1.02–1.26)	- 0.7 5.1
Water pipe	47/53 (89) 182/194 (94) 299/311 (96)	0.030	(ref) 1.03 (0.93–1.14) 1.06 (0.95–1.17)	1 (ref) 1.05 (0.92–1.20) 1.09 (0.97–1.22)	1 (ref) 1.06 (0.94–1.20) 1.09 (0.97–1.21)	- 2.0 2.7
Any tobacco use	33/51 (65) 121/181 (67) 232/302 (77)	0.040	1 (ref) 1.03 (0.88–1.21) 1.19 (0.03–1.37)	1 (ref) 1.16 (0.93–1.45) 1.27 (1.07–1.51)	1 (ref) 1.08 (0.84–1.38) 1.21 (1.05–1.39)	- 1.1 5.5

^1^Adjusted for student’s gender, student’s smoking friends, friends enjoying school and performance of friends at school, parental education, parental occupation, and parental smoking

^2^Adjsuted for 1 + school level variables: average merit score, school average parental education, teacher density, proportion of teachers with university education, implementation of other prevention programs, implementation of other health promotion programs, and implementation level of all other T-Duo components using the three categories (poor, satisfactory, per protocol)

^3^Bayes factor less than 1/3 indicates evidence for the 0 hypotheses (no effect), greater than 3 indicates evidence for an effect (alternative hypothesis), and any value between 1/3 and 3 indicates that the findings are inconclusive (Dienes, 2014)

^4^Unstable contract refers to students that wrote the contract only for 1 or 2 year(s), or switched contract partner at least once

Contrasting a stable 3-year contract vs no contract (Table 3) yielded larger effect estimates compared to the estimates from the binary categorization (ever vs never signed a contract). The adjusted RR to have remained a non-cigarette smoker over the 3-year period was 1.15 (1.04–1.26) among students that were in a stable 3-year contact compared to students without a contract at all (Table 3). Risk estimates for students in an unstable contract compared to no contract were inconclusive (Table 3). For all outcomes of the unstable contract group, the confidence intervals were wide, and the estimates were therefore compatible with the null hypothesis of no effect (Table 3). Absolute and relative risk estimates from the fully adjusted model (adjusted for both individual covariates and school-level covariates) were similar to the unadjusted estimates in all analyses (Tables 2 and 3).

### Sensitivity Analyses

Sensitivity analysis 1, additionally including students that answered at baseline and follow-up 3 (*n* = 78) yielded slightly weaker associations compared to the main analysis, and most estimates were inconclusive (Appendix 1, Table 6). Sensitivity analysis 2, assessing the effect at 2-year follow up yielded effect estimates that were slightly smaller but consistent with the 3-year follow-up. The effect estimates at 2-year follow-up (RR_non-smoker_ = 1.07 (0.99–1.16)) and 3-year follow-up (RR_non-smoker_ = 1.15 (1.04–1.26)) are larger than the minimal effect sizes observed in the previously published ITT analysis at 2-year follow-up (RR_non-smoker_ = 1.03 (0.98–1.08)) (Beeres et al., 2022) (Appendix 1, Table 7 and 8). Sensitivity analysis 3 applying multilevel ordinal logistic regression showed that largest preventive effects were seen among students that formed a stable 3-year contract compared to students with an unstable contract or without a contract at all (Appendix 1, Table 9).

## Discussion

We evaluated whether a 3-year commitment to remain tobacco free in the form of a contract between a child and a significant adult was associated with the probability to refrain from initiating tobacco use among high school students targeted by a comprehensive school-based intervention. We found that the uptake of cigarette smoking was indeed lower among students who built a contract with a significant adult, compared to students who did not sign a contract at all. In addition, we observed a dose–response effect, as the uptake of all types of tobacco products was lower among students who built a stable contract with a significant adult compared to students without a contract at all, while the findings for students in an intermediate position (signed a contract, but it was not sustained over time and/or with the same adult) were inconclusive.

The results show that being in a contract entails in any case a desirable effect on onset of tobacco use, but that the substantial benefit is linked to long duration and sustained partnership. This indicates that the contract is a significant “ingredient” in the 6-component T-Duo intervention. The advantage of this core component of T-Duo when adherence is complete, i.e., being in a stable 3-year contact, translates in nine students who needed to be in this condition to prevent one student from initiation of cigarette use, compared to ten students when assessing any contract length (ever vs never contract). From a public health perspective, nine to ten students needing to adhere to the intervention in order to prevent one from tobacco initiation can be considered a satisfactory and relevant goal, particularly in contexts of relatively low and decreasing rates of tobacco (cigarette) uptake among adolescents (Thomas et al., 2015). Bayes factors indicated evidence in favor of the alternative hypothesis for students in a stable contract for onset of cigarette smoking, e-cigarette use, and the combined measure any type of tobacco use but not for the secondary outcomes related to more advanced use of cigarettes or for the use of other tobacco products.

For the students who were in an unstable contract, we observed an indication of positive effects for most outcomes although the effect estimates for all outcomes were inconclusive. Reasons for not being in a contract or for not being able to hold a stable contract may be linked to the projected outcome (e.g., own or significant adult smoking initiation) but also to family events such as divorce, death, or other disruptive situations. Unfortunately, we did not have information on family events during follow-up, and it is possible that these factors contributed to relatively small effect sizes in the unstable contract group.

Only 6% of the students in the stable contract group ever tried any type of tobacco at baseline and were excluded for the respective outcome, which contrasts with the larger proportion of 11% tobacco users at baseline in the other two contract groups. This shows that smokers were less likely to enter a stable 3-year contract. A Danish study also reported that students who smoked cigarettes were less likely to enter a contract in the first place (Bast et al., 2021). However, most of the questioned participants stated that the reason for not signing a contract was because they were never invited to sign a contract, alternatively that there was no specific reason (“I just didn’t do it”) (Bast et al., 2021). Both in our study and in the Danish study, girls were more likely to be in a (stable) contract, while in Sweden, the smoking prevalence is higher among girls compared to boys (Bast et al., 2021; Beeres et al., 2022). Multiple unknown factors other than tobacco use and gender might have affected the probability to enter and remain in a contract, but studies are rare, and apart from this single Danish study, factors related to participation in such a contract are largely unexplored (Bast et al., 2021).

The tobacco-free contract is the core component of a very comprehensive program, and how the six components of this program interacted with each other is not known. Assessment of implementation of the program, in which schools were categorized based on monthly reports of completed activities by the school’s contact persons, showed that the proportion of students that signed a contract was related to the overall degree of implementation of the intervention at the school level (Nilsson et al., 2022). In the schools that implemented T-Duo according to protocol, 88% of students in the analytical sample wrote a contract (41% unstable, 47% stable) compared to 72% (44% unstable, 28% stable) of students in schools that were classified as low implementation (Nilsson et al., 2022). However, controlling for implementation level did not change the relative risk estimates (Table 2).

This could have multiple explanations, among other things higher implementation of other intervention components among high-risk schools or lower effect of the contract among high implementation schools. The difference in the cumulative incidence of uptake of tobacco products (for instance, e-cigarette was common while smoking a whole cigarette, regular cigarette use, and water pipe use were rare for all three groups) might have resulted in minimal effect sizes for the rarer outcomes, reflected in the higher risk estimates for all outcomes with a prevalence of never triers below 90%. In both the previous ITT evaluation and the current study, the largest effects were observed for the prevention of cigarette use, e-cigarette uptake, and any type of tobacco product, which increases the robustness of these findings. However, there are other explanatory factors that affected the effect sizes in both studies differently. Where in the ITT poor uptake and implementation is a likely explanation that contributed to the small effect sizes, in this AT analysis, the abovementioned selection of participants at lower risk of tobacco initiation might have altered the observed effect sizes.

### Strengths and Limitations

To the best of our knowledge, this is the first study that rigorously evaluated with an “as-treated” approach the effect of a public commitment to remain tobacco free through a formal contract between an adolescent and an adult. This is somewhat surprising because interventions based on a public commitment are widespread and recommendations are issued to assess the effect of the core component of multi-component interventions separately (Hernán & Hernández-Díaz, 2012). We applied state-of-the-art methodology in the data analysis, considering the cluster-based design and adjusting for confounders derived from an a priori causation frame. Also, we provided both absolute and relative measures of the program’s effect to improve the usefulness of the results for a wide audience including health practitioners and policy makers. Adjustment for individual-level and school-level covariates did not change the magnitude of the associations, which implies that the findings were robust regarding the effect of a 3-year stable contract on smoking initiation. From a public health perspective, this is of interest in the light of a potential dissemination of the program. The relatively long follow-up time of nearly 3 years covers a large part of the risk period for cigarette uptake and indicates that sustained intervention efforts are part of the effects of this and similar programs.

This study also had several limitations. First and foremost, this is a secondary analysis of a c-RCT. Although schools were randomly assigned to the intervention arm, in this analysis, we used adherence to contract status, which was not following a random assignment and implied non-comparability of groups and consequent bias (Ranganathan et al., 2015). We used directed acyclic graphs (DAGs) and causal assumptions to identify potential confounding factors. However, residual confounding of unmeasured characteristics is possible. Although we excluded all baseline users of tobacco products, students in the contract group might still be a selected sample of participants that were more likely to remain tobacco free during the study period. For example, among all students that never tried any tobacco product at baseline, students that reported to have smoking friends were less than half as likely to be in a stable contract compared to students without smoking friends. As mentioned earlier, unstable family conditions might relate to not signing a contract or being in an unstable contract, as well as to initiation of tobacco use, i.e., they can act as confounders. Unfortunately we were not able to control for these factors.

The selective participation of schools, the analytical sample consisting of full compliers to all three follow-up surveys, and the selection of “low-risk” participants may all impair the generalizability of the results to the whole source population and to high-risk populations. However, on student level, the sensitivity analysis did not show substantially different effect estimates when also including students that answered only at baseline and follow-up 3. In addition, the last 1.5 years of follow-up period occurred during the COVID-19 pandemic, and the risk behavior of teenagers might not be representative for other years. For example, the larger amount of time spent at home, the reduced contact with peers at school, and knowledge of smoking as a potential risk factor for severe COVID-19 might have impacted the student’s risk behavior (Chaffee et al., 2021; Lundahl & Cannoy, 2021).

Tobacco use was self-reported and might be underreported. Specifically, another program component was an annual affirmation of non-smoking status and lottery draw among students with a contract at the end of each school year. This may have created an incentive to underreport tobacco use to a greater extent among students who had signed a contract, thus resulting in information bias (Chaffee et al., 2021; Thomas et al., 2015).

The effect estimates observed in this study at follow-up 2 are larger than the minimal effect sizes observed in the previously published ITT analysis which eventually should guide public health decisions (Ranganathan et al., 2015). Since the ITT analysis had a follow-up time of 2 years, only the estimates from the sensitivity analysis that report on the 2-year follow-up are directly comparable.

For both the ITT and this AT analysis, the prevalence of ever triers of the respective tobacco products was related to the observed effect estimates and might explain the small effect estimates for these outcomes in both evaluations.

To conclude, being in a stable tobacco-free pair seems to exert a preventive effect on the uptake of tobacco use among Swedish adolescents over 3 school years. The program was particularly effective in the prevention of ever-trying conventional cigarette use and of overall tobacco use. The current findings apply to a selected sample of schools and students and further evaluations of potential adverse effects as well as of the effect of the program on high-risk populations is needed before active dissemination. Reducing socioeconomic inequities in tobacco-related morbidity and mortality is a priority, particularly in the context of high-income countries as Sweden where cigarette use declines but also segregates among sub-groups of the populations that are already economically and/or socially disadvantaged (Loring, 2014).

### Supplementary Information

Below is the link to the electronic supplementary material.

Supplementary file1 (DOCX 30 KB)

## Appendix 1: Absolute Risk Difference (ARD) and Number Needed to Treat (NNT) corresponding to Tables 2 and 3 in the main manuscript

**Table 4:** Absolute risk differences and Number Needed to Treat for the primary and secondary outcomes at the end of grade 9 among students in the T-Duo program who were never users of the corresponding type of tobacco at baseline, by binary contract status

	**Contract status**		**Absolute risk difference (ARD)**			
Not started with	Never contract (ref) Ever contract *Proportion non-user’s follow-up 3*	*ICC*	**Risk difference**—**Model 0** (crude) % Diff(95%CI)	**Risk difference—Model 1** (adjusted individual covariates)^1^ % Diff (95%CI)	**Risk difference—Model 2** (model 1 + school covariates)^2^ % Diff (95% CI)	**NNT**
**Primary outcome**						
Cig. smoking (even a few puffs)	41/54 (76) 427/514 (83)	0.018	1 (ref) 9.00 (− 0.00 to 18.0)	1 (ref) 11.2 (− 0.6 to 23.1)	1 (ref) 10.0 (0.00 to 20.01)	- 10
**Secondary outcomes**						
Cig. smoking (whole cigarette)	46/53 (87) 469/514 (91)	0.022	1 (ref) 6.90 (0.45 to 13.4)	1 (ref) 9.17 (1.03 to 17.3)	1 (ref) 10.1 (1.18 to 19.0)	- 10
Regular cig. smoking	48/54 (89) 505/517 (98)	0.004	1 (ref) 9.43 (− 0.89 to 19.7)	1 (ref) 9.48 (− 3.30 to 22.3)	1 (ref) 10.2 (− 3.16 to 23.6)	- 10
Snus use	40/55 (73) 402/506 (79)	0.037	1 (ref) 8.83 (− 6.81 to 24.5)	1 (ref) 14.3 (− 5.73 to 34.2)	1 (ref) 11.04 (− 6.47 to 28.6)	- 9
Reg. snus use	50/57 (88) 494/519 (95)	0.000	1 (ref) 8.17 (2.69 to 13.6)	1 (ref) 8.25 (1.25 to 15.2)	1 (ref) 6.84 (− 1.39 to 15.1)	¨ 15
E-cig use	40/50 (80) 424/492 (86)	0.064	1 (ref) 7.43 (− 1.36 to 16.2)	1 (ref) 8.90 (− 1.16 to 19.1)	1 (ref) 7.49 (− 3.78 to 18.8)	- 13
Water pipe	47/53 (89) 481/505 (95)	0.030	1 (ref) 7.15 (− 3.42 to 17.7)	1 (ref) 11.1 (− 1.97 to 24.1)	1 (ref) 11.8 (− 1.61 to 2.52)	- 8
Any tobacco use	41/51 (65) 353/483 (73)	0.040	1 (ref) 12.1 (− 1.17 to 25.5)	1 (ref) 19.7 (1.65 to 37.8)	1 (ref) 15.0 (− 1.66 to 31.5)	- 7

^1^Adjusted for student’s gender, student’s smoking friends, friends enjoying school and performance of friends at school, parental education, parental occupation, and parental smoking

^2^Adjsuted for 1 + school level variables: average merit score, school average parental education, teacher density, proportion of teachers with university education, implementation of other prevention programs, implementation of other health promotion programs, and implementation level of all other T-Duo components using the three categories (poor, satisfactory, per protocol)

**Table 5:** Absolute Risk Differences and Number Needed to Treat for the primary and secondary outcomes at the end of grade 9 among students in the T-Duo program who were never users of the corresponding type of tobacco at baseline, by contract status

	**Contract status**		**Absolute Risk Difference (ARD)**			
Not started with	**No contract (ref)** **Unstable contract** **Stable contract** *Proportion non-user’s follow-up 3*	*ICC*	**Risk difference**—**Model 0** (crude) % Diff (95%CI)	**Risk difference—Model 1** (adjusted individual covariates)^1^ % Diff (95%CI)	**Risk difference—Model 2** (adjusted individual + school covariates)^2^ % Diff (95% CI)	**NNT**
Primary outcome						
Cig smoking (even a few puffs)	41/54 (76) 152/197 (77) 275/317 (87)	0.018	0 (ref) 1.6 (− 9.2 to 12.4) 13.3 (4.1 to 22.5)	0 (ref) 5.1 (− 9.0 to 19.4) 14.4 (2.7 to 26.2)	0 (ref) 3.1 (− 10.4 to 16.6) 13.4 (3.8 to 23.0)	*-* 33 7
Secondary outcomes						
Cig. smoking (whole cigarette)	46/53 (87) 172/196 (88) 297/318 (93)	0.022	0 (ref) 1.1 (− 7.0 to 9.2) 7.0 (1.1 to 13.6)	0 (ref) 5.0 (− 5.8 to 15.6) 8.8 (0.0 to 16.8)	0 (ref) 5.1 (− 6.6 to 16.7) 10.4 (1.3 to 19.4)	- 20 10
Regular cig. smoking	48/54 (89) 189/198 (95) 316/319 (99)	0.004	0 (ref) 5.7 (− 2.6 to 14.1) 9.0 (0.9 to 17.0)	0 (ref) 5.6 (− 4.8 to 15.9) 8.7 (− 0.6 to 17.9)	0 (ref) 5.4 (− 4.2 to 15.0) 6.8 (− 1.7 to 15.3)	- 18 15
Snus use	40/55 (73) 147/194 (76) 255/312 (82)	0.037	0 (ref) 4.1 (− 13.8 to 22.0) 11.7 (− 5.0 to 28.3)	0 (ref) 10.9 (− 12.4 to 34.3) 16.1 (− 4.2 to 36.3)	0 (ref) 5.7 (− 17.8 to 29.2) 13.8 (− 3.2 to 30.8)	- 18 7
Reg. snus use	50/57 (88) 182/202 (90) 312/317 (98)	0.000	0 (ref) 2.7 (− 3.1 to 8.5) 11.5 (5.8 to 17.2)	0 (ref) 1.6 (− 6.7 to 9.9) 12.0 (5.3 to 18.7)	0 (ref) − 0.1 (− 10.0 to 8.5) 11.0 (0.3 to 19.0)	- (-133) 9
E-cig. use	40/50 (80) 149/183 (81) 275/309 (89)	0.064	0 (ref) 1.8 (− 9.5 to 13.1) 10.7 (1.2 to 20.1)	0 (ref) 3.1 (− 11.3 to 17.4) 11.9 (0.2 to 22.1)	0 (ref) 0.0 (− 17.6 to 18.1) 10.7 (0.7 to 20.7)	- 393 9
Water pipe	47/53 (89) 182/194 (94) 299/311 (96)	0.030	0 (ref) 5.6 (− 5.3 to 16.6) 8.1 (− 2.5 to 18.7)	0 (ref) 9.2 (− 5.1 to 23.5) 12.2 (− 0.6 to 25.0)	0 (ref) 10.7 (− 4.2 to 25.5) 12.4 (− 0.6 to 25.4)	- 9 8
Any tobacco use	33/51 (65) 121/181 (67) 232/302 (77)	0.040	0 (ref) 3.3 (− 12.7 to 19.3) 17.2 (2.7 to 31.6)	0 (ref) 13.9 (− 9.0 to 36.8) 22.7 (4.4 to 40.9)	0 (ref) 3.2 (− 18.5 to 32.9) 18.3 (2.4 to 34.2)	- 14 5

^1^Adjusted for student’s gender, student’s smoking friends, friends enjoying school and performance of friends at school, parental education, parental occupation, and parental smoking

^2^Adjsuted for 1 + school level variables: average merit score, school average parental education, teacher density, proportion of teachers with university education, implementation of other prevention programs, implementation of other health promotion programs, and implementation level of all other T-Duo components using the three categories (poor, satisfactory, per protocol)

## **Sensitivity Analysis 1:** Binary Categorization of Contract Status and Inclusion of Students with Missing Information on Contract Status at Follow-up 1 and 2

**Table 6:** Risk Ratios to remain tobacco free at the end of grade 9 among students that were never users of the corresponding type of tobacco at baseline and answered at baseline and grade 9, by contract status (ever vs never contract)

	**Contract status**		**Risk ratio’s (95% CI) to remain a non-user after 3 school years among non-users at baseline**			
Not started with	**Never contract (ref)** **Ever contract** *Proportion non-user’s follow-up 3*	ICC	**Model 0—crude** RR (95% CI)	**Model 1—adjusted for individual covariates** ^**1**^ RR (95% CI)	**Model 2—adjusted for individual + school covariates** ^**2**^ RR (95% CI)	**Bayes factor** ^**3**^ BF_10_
**Primary outcome**						
Cig. smoking (even a few puffs)	63/83 (76) 465/563 (83)	0.018	1 (ref) 1.09 (0.98–1.21)	1 (ref) 1.10 (0.96–1.25)	1 (ref) 1.09 (0.97–1.23)	- 1.9
**Secondary outcomes**						
Cig. smoking (whole cigarette)	70/82 (85) 510/563 (91)	0.022	1 (ref) 1.06 (0.99–1.14)	1 (ref) 1.09 (1.00–1.18)	1 (ref) 1.11 (1.00–1.23)	- 2.4
Regular cig. smoking	75/83 (90) 552/566 (98)	0.004	1 (ref) 1.10 (0.99–1.22)	1 (ref) 1.10 (0.97–1.25)	1 (ref) 1.09 (0.98–1.21)	- 2.4
Snus use	60/84 (73) 437/555 (79)	0.037	1 (ref) 1.10 (0.95–1.28)	1 (ref) 1.19 (0.93–1.30)	1 (ref) 1.08 (0.91–1.27)	- 1.2
Reg. snus use	76/87 (87) 542/569 (95)	0.000	1 (ref) 1.09 (1.03–1.16)	1 (ref) 1.09 (1.02–1.17)	1 (ref) 1.08 (0.99–1.17)	¨ 3.3
E-cig. use	61/76 (80) 466/526 (87)	0.064	1 (ref) 1.08 (0.99–1.18)	1 (ref) 1.10 (1.00–1.21)	1 (ref) 1.09 (0.98–1.21)	- 2.3
Water pipe	75/582 (91) 526/553 (95)	0.030	1 (ref) 1.04 (0.97–1.11)	1 (ref) 1.08 (0.98–1.20)	1 (ref) 1.10 (0.98–1.23)	- 2.3
All tobacco use	50/76 (66) 387/526 (74)	0.040	1 (ref) 1.12 (0.97–1.29)	1 (ref) 1.18 (1.00–1.39)	1 (ref) 1.14 (0.98–1.33)	- 2.4

^1^Adjusted for student’s gender, student’s smoking friends, friends enjoying school and performance of friends at school, parental education, parental occupation, and parental smoking

^2^Adjsuted for 1 + school level variables: average merit score, school average parental education, teacher density, proportion of teachers with university education, implementation of other prevention programs, implementation of other health promotion programs, and implementation level of all other T-Duo components using the three categories (poor, satisfactory, per protocol)

^3^Bayes factor less than 1/3 indicates evidence for the 0 hypotheses (no effect), greater than 3 indicates evidence for an effect (alternative hypothesis), and any value between 1/3 and 3 indicates that the findings are inconclusive (Dienes, 2014)

## **Sensitivity Analysis 2: **Main Analysis (as in Tables 2 and 3 of the Manuscript) at 2-year follow-up (Interim Analysis to be Compared with Previously Published ITT Results)

**Table 7:** Risk Ratios to remain tobacco free at the end of grade 8 among students that were never users of the corresponding type of tobacco at baseline and participated in the two follow-up surveys by contract status (ever vs never signed a contract)

Not started with	Never contract (ref) Ever contract *Proportion non-user’s follow-up 2*	ICC	**Model 0—crude** RR (95% CI)	**Model 1—adjusted for individual covariates** ^**1**^ RR (95% CI)	**Model 2—adjusted for individual + school covariates** ^**2**^ RR (95% CI)	**Bayes factor** ^**3**^ BF_10_
**Primary outcome**						
Cig. smoking (even a few puffs)	46/54 (85) 461/515 (90)	0.020	1 (ref) 1.05 (0.97–1.14)	1 (ref) 1.06 (0.97–1.15)	1 (ref) 1.07 (0.99–1.16)	- 2.4
**Secondary outcomes**						
Cig. smoking (whole cigarette)	50/55 (91) 469/514 (95)	0.000	1 (ref) 1.04 (0.96–1.12)	1 (ref) 1.04 (0.97–1.12)	1 (ref) 1.05 (0.96–1.13)	- 1.1
Regular cig. smoking	51/55 (93) 514/516 (100)	0.000	1 (ref) 1.07 (0.98–1.18)	1 (ref) 1.07 (0.96–1.19)	1 (ref) 1.07 (0.95–1.20)	- 1.4
Snus use	43/54 (80) 463/507 (91)	0.008	1 (ref) 1.15 (1.96–1.24)	1 (ref) 1.15 (1.01–1.31)	1 (ref) 1.17 (1.02–1.33)	- 6.3
Reg. snus use	53/56 (95) 511/519 (98)	0.000	1 (ref) 1.04 (0.99–1.10)	1 (ref) 1.03 (0.97–1.08)	1 (ref) 1.03 (0.98–1.09)	¨ 0.9
E-cig. use	44/51 (86) 462/493 (94)	0.003	1 (ref) 1.09 (1.03–1.14)	1 (ref) 1.09 (1.01–1.18)	1 (ref) 1.09 (1.00–1.18)	- 3.7
Water pipe	53/56 (95) 497/507 (98)	0.030	1 (ref) 1.04 (0.98–1.10)	1 (ref) 1.05 (0.98–1.12)	1 (ref) 1.05 (0.98–1.12)	- 1.2
Any tobacco use	41/52 (78) 414/482 (86)	0.012	1 (ref) 1.09 (0.98–1.21)	1 (ref) 1.14 (1.0–1.28)	1 (ref) 1.13 (1.02–1.26)	- 7.5

^1^Adjusted for student’s gender, student’s smoking friends, friends enjoying school and performance of friends at school, parental education, parental occupation, and parental smoking

^2^Adjsuted for 1 + school level variables: average merit score, school average parental education, teacher density, proportion of teachers with university education, implementation of other prevention programs, implementation of other health promotion programs, and implementation level of all other T-Duo components using the three categories (poor, satisfactory, per protocol)

^3^Bayes factor less than 1/3 indicates evidence for the 0 hypotheses (no effect), greater than 3 indicates evidence for an effect (alternative hypothesis), and any value between 1/3 and 3 indicates that the findings are inconclusive (Dienes, 2014)

**Table 8:** Risk Ratios to remain tobacco free at the end of grade 8 among students in the T-Duo program who were never users of the corresponding type of tobacco at baseline, by contract status (3 categories)

	**Contract status**		**Risk ratios (95% CI) to remain a non-user after 2 school years among non-users at baseline**			
Not started with	**Never contract (ref)** **Unstable contract** ^**4**^ **Stable 2-year contract** *Proportion* *Non-users at follow-up 2*	ICC	**Model 0—crude** RR (95% CI)	**Model 1—adjusted for individual covariates** ^**1**^ RR (95% CI)	**Model 2—adjusted for individual + school covariates** ^**2**^ RR (95% CI)	**Bayes factor** ^**3**^ (BF_10_)
**Primary outcome**						
Cig. smoking (even a few puffs)	46/54 (85) 168/196 (86) 293/319 (92)	0.020	1 (ref) 1.01 (0.92–1.10) 1.08 (0.99–1.17)	1 (ref) 1.02 (0.91–1.13) 1.08 (0.99–1.18)	1 (ref) 1.01 (0.91–1.12) 1.10 (1.02–1.20)	- 0.6 8.2
**Secondary outcomes**						
Cig. smoking (whole cigarette)	50/55 (91) 178/196 (91) 308/318 (97)	0.000	1 (ref) 1.00 (0.92–1.09) 1.07 (0.98–1.15)	( 1.00 (0.92–1.09) 1.06 (0.98–1.15)	1 (ref) 0.99 (0.91–1.09) 1.08 (0.98–1.18)	- 0.4 1.6
Regular cig. smoking	51/55 (93) 195/197 (99) 319/319 (100)	0.000	1 (ref) 1.07 (0.97–1.17) 1.08 (0.99–1.18)	1 (ref) 1.06 (0.95–1.19) 1.07 (0.96–1.20)	1 (ref) 1.07 (0.95–1.20) 1.08 (0.96–1.21)	- 1.4 1.6
Snus use	43/54 (80) 174/194 (90) 289/313 (92)	0.008	1 (ref) 1.13 (1.02–1.23) 1.16 (1.06–1.27)	1 (ref) 1.13 (0.98–1.31) 1.16 (1.03–1.32)	1 (ref) 1.15 (0.98–1.34) 1.19 (1.06–1.35)	- 2.9 2.6
Reg. snus use	53/56 (95) 193/200 (97) 318/319 (100)	0.000	1 (ref) 1.02 (0.96–1.08) 1.04 (1.04–1.09)	1 (ref) 1.02 (0.95–1.09) 1.02 (0.99–1.10)	1 (ref) 1.02 (0.96–1.08) 1.05 (1.00–1.11)	- 1.0 2.5
E-cig. use	44/51 (86) 167/184 (91) 295/309 (95)	0.003	(ref) 1.05 (0.99–1.11) 1.11 (1.03–1.19)	(ref) 1.06 (0.97–1.16) 1.12 (1.02–1.22)	1.04 (0.94–1.15) 1.11 (1.02–1.21)	- 1.6 7.2
Water pipe	53/56 (95) 187/193 (97) 310/314 (99)	0.030	(ref) 1.03 (0.96–1.11) 1.05 (0.98–1.12)	(ref) %1.%2 0.96–1.14) 1.06 (0.98–1.15)	1 (ref) 1.05 (0.96–1.15) 1.06 (0.99–1.14)	- 1.2 2.1
Any tobacco use	41/52 (65) 149/180 (83) 265/302 (88)	0.012	1 (ref) %1.%2 0.96–1.14) 1.11 (0.98–1.26)	1. (ref) 1.12 (0.99–1.26) 1.17 (1.01–1.36)	1 (ref) 1.09 (0.96–1.23) 1.17 (1.01–1.34)	- 1.8 5.9

^1^Adjusted for student’s gender, student’s smoking friends, friends enjoying school and performance of friends at school, parental education, parental occupation, and parental smoking

^2^Adjsuted for 1 + school level variables: average merit score, school average parental education, teacher density, proportion of teachers with university education, implementation of other prevention programs, implementation of other health promotion programs, and implementation level of all other T-Duo components using the three categories (poor, satisfactory, per protocol)

^3^Bayes factor less than 1/3 indicates evidence for the 0 hypotheses (no effect), greater than 3 indicates evidence for an effect (alternative hypothesis), and any value between 1/3 and 3 indicates that the findings are inconclusive (Dienes, 2014) ^4^Unstable contract refers to students that wrote the contract only for 1 or 2 year(s), or switched contract partner at least once

## **Sensitivity Analysis 3**: Multilevel Ordered Logistic Regression, Assessing the Odd Ratio´s to Remain Tobacco Free at the End of Grade 9 Among Students in the T-Duo Program who were never Users of the Corresponding Type of Tobacco at Baseline, by Contract Status

**Table 9:** Odds Ratios to remain tobacco free at the end of grade 9 among students in the T-Duo program who were never users of the corresponding type of tobacco at baseline, by contract status

	**Contract status**		**Odds ratios (95% CI) to remain a non-user after 3 school years among non-users at baseline**		
	**No contract (ref)** **Unstable contract** **Stable contract** *Proportion non-users follow-up 3*	ICC	**Model 0—crude** OR (95% CI)	**Model 1—adjusted for individual covariates** ^**1**^ OR (95% CI)	**Model 2—adjusted for individual + school covariates** ^**2**^ OR (95% CI)
**Primary outcome**					
Cig. smoking (even a few puffs**)**	41/54 (76) 152/197 (77) 275/317 (87)	0.018	1 (ref) 1.08 (0.69–1.70) 2.18 (1.44–3.30)	1 (ref) 1.42 (0.66–3.05) 2.45 (1.20–5.02)	1 (ref) 1.23 (0.57–2.66) 2.31 (1.25–4.27)
**Secondary outcomes**					
Cig. smoking (whole cigarette)	46/53 (87) 172/196 (88) 297/318 (93)	0.022	1 (ref) 1.31 (0.66–2.60) 2.97 (1.59–5.54)	1 (ref) 1.80 (0.68–4.78) 3.62 (1.73–7.59)	1 (ref) 1.95 (0.67–5.67) 4.07 (1.82–9.07)
Regular cig. smoking	48/54 (89) 189/198 (95) 316/319 (99)	0.004	1 (ref) 2.66 (0.76–9.27) 13.56 (4.53–40.6)	1 (ref) 3.55 (0.51–24.5) 23.0 (7.19–73.68)	1 (ref) 3.16 (0.39–25.54) 19.3 (3.00–123.7)
Snus use	40/55 (73) 147/194 (76) 255/312 (82)	0.037	1 (ref) 1.16 (0.56–2.39) 1.67 (0.85–3.27)	1 (ref) 1.50 (0.58–3.90) 1.94 (0.91–4.11)	1 (ref) 1.30 (0.54–3.11) 1.79 (0.99–3.23)
Reg. snus use	50/57 (88) 182/202 (90) 312/317 (98)	0.000	1 (ref) 1.43 (0.66–3.08) 8.22 (2.47–27.3)	1 (ref) 1.63 (0.58–4.60) 14.9 (3.14–70.4)	1 (ref) 0.99 (0.30–3.30) 10.62 (2.68–42.18)
E-cig. use	40/50 (80) 149/183 (81) 275/309 (89)	0.064	1 (ref) 1.17 (0.60–2.27) 2.19 (0.90–5.33)	1 (ref) 1.31 (0.57–2.98) 2.48 (1.13–5.43)	1 (ref) 1.17 (0.48–2.88) 2.30 (1.04–5.06)
Water pipe	47/53 (89) 182/194 (94) 299/311 (96)	0.030	(ref) 1.87 (0.48–7.32) 3.62 (1.02–12.9)	1 (ref) 2.01 (0.32–12.6) 6.23 (1.40–27.64)	1 (ref) 2.14 (0.23–19.90) 6.80 (1.04–44.26)
Any tobacco use	33/51 (65) 121/181 (67) 232/302 (77)	0.040	1 (ref) 1.12 (0.64–1.98) 1.83 (1.14–2.94)	1 (ref) 1.64 (0.69–3.87) 2.31 (1.26–4.26)	1 (ref) 1.31 (0.54–3.18) 1.95 (1.12–3.40)

^1^Adjusted for student’s gender, student’s smoking friends, friends enjoying school and performance of friends at school, parental education, parental occupation, and parental smoking

^2^Adjsuted for 1 + school level variables: average merit score, school average parental education, teacher density, proportion of teachers with university education, implementation of other prevention programs, implementation of other health promotion programs, and implementation level of all other T-Duo components using the three categories (poor, satisfactory, per protocol)

## Appendix 2: Questionnaire item exposure, primary and secondary outcomes and covariates

**Exposure contract status:** Are you part of a tobacco-free pair (wrote the contract)? “Yes, No.”

(Follow-up 2 only): Did you change contract partner since you wrote the contract for the first time? “Yes; No.”

**Primary outcome:** Onset of cigarette smoking was self-reported by answering the question: (1) “Have you ever tried smoking a cigarette, even if it was just a few puffs?” “No, never tried; Yes”. Adolescents that answered “No, never tried” were classified as never cigarette smokers (even not a few puffs).

**Secondary outcomes***:* Secondary outcomes were related to.

- Advanced use of cigarettes, assessed through the questions (2) “Have you ever smoked a whole cigarette?” “Yes/ No, never smoked/ No, never a whole cigarette” And “Have you ever smoked cigarettes regularly? (defined as at least once a week for at least 3 consecutive months).” “Yes/No, never smoked regularly/Never tried smoking.”
- The onset of use of other types of tobacco/nicotine, i.e., snus, e-cigarettes and/or water pipe (3). The initial use of other types of tobacco/e-cigarettes was assessed in the same questionnaire through the questions: “Have you ever tried snus? Have you ever tried e-cigarettes? Have you ever tried waterpipe smoking?” with response options corresponding to those for cigarette smoking. An affirmative answer to any of these questions was categorized as “Having tried any type of tobacco product.” The use of snus was also assessed as regular use, with a question similar to cigarette smoking.

## Covariates

**Student questionnaire:**

**Sex:** Are you? “Girl; Boy; Other; Don’t want to say”.

**Smoking friends:** Do some of the following person smoke? e. Some of your closest friends’? “Smoke daily; Smoke sometimes; Don’t smoke; Don’t know; Don’t see them/Don’t have them.”

**Table:**

Do some of the following person smoke?			
	0	1	
e. Some of your closest friends’?	“Don’t smoke” “Don’t have them”	“Smoke daily” Smoke sometimes	“I don’t know”

**Friends enjoyment/performance school:** Of your friends which whom you spent most of your time with. How many enjoy school? How many perform well at school? “No one,” “less than half,” “about half,” “more than half,” “all,” “I don’t know.”

**Table:**

Of your friends which whom you spent most of your time with;			
	0	1	
How many enjoy school?	“No one,” “less than half,” “about half”	“more than half,” “all”	“I don’t know”
How many perform well at school?	“No one,” “less than half,” “about half”	“more than half,” “all”	“I don’t know”

**Parental questionnaire (baseline)**

How old are you ?* ….*

What is your highest completed education? “Pre high school shorter than 9 years. Pre high school 9 years. High school shorter than 3 years. High school 3 years. Higher education (after high school) shorter than 3 years. Higher education longer than 3 years. Doctorand/Equivalent. Other education ()”.

What is your current employment status? “Fixed contract. Temporary contract. Self-employed. Sick leave (more than 30 days). Early pensioner/include sick leave compensation or activity compensation; Pensioner; Studying; Parental leave or other leave); Unemployed/Job Seeker; Informal work at home/ responsible household; Other employment(….).”

**TOPAS schools (questionnaire to contract person TOPAS schools)**

Is the school currently participating in any ANDT prevention program and/or activity? “No. Yes. Please enter name and briefly describe its contents: …”.

Does the school participate in other health promotion or prevention programs and/or activities? “No. Yes. Please enter name and briefly describe its contents: …”.

Other school level variables were collected from the national school database.
